# Notes and News

**Published:** 1887-10-15

**Authors:** 


### Notes and News.

Collected and Communicated.

The harvest thanksgiving and the Hospital Sunday collection have taken place conjointly in the various places of worship at Cirencester this year. The collection at the parish church amounted to the handsome sum of £38 0s. 3d.

A contract for the erection of a new fever hospital in the Isle of Man has been again entered into. The total cost of the erection is to be £2,130.

£5,804 4s. 8d. is the amount of legacies and donations given to the Edinburgh Royal Infirmary during the last half-year. Of this sum John Scott, labourer, of Loanhead, near Dollar, contributed no less than £136 9s. 8d.

Twenty pounds an acre is the amount asked for ground for a site of the proposed hospital at Duddingston, near Edinburgh. Half an acre only is required, and if the proprietor can afford to sell it for the small sum of ten pounds, it is not improbable that he can afford to give it outright. It is, at any rate, worth a consideration.

A new orthopædic hospital has been opened at Hull. Although the population of that prosperous seaport is close upon 200,000, there has hitherto been no institution of the kind in the town. Mrs. Halden, the lady superintendent of the Hull Nursing Home, has been appointed matron, Mr. Hagyard, honorary surgeon, and Mr. John O'Brien, honorary secretary. One feature of the hospital is the abolition of all recommendation forms.

The new hospital at Hastings was opened on the 13th. The medical staff, as well as the committee, have personally and by their influence very materially helped the building fund. This was perhaps to be expected, though it is not the less praiseworthy on that account. It is said that the names of many well-to-do and liberal people have not yet appeared on the list of donors. Surely, if doctors, surgeons, and members of committee give both time and money to the institution, other portions of the community equally prosperous should be prompt with liberal subscriptions. It is highly discreditable to a large part of the public that they leave all good works to be done by a very few.

The latest addition to the Library of The Hospitals Association for reference by members, consists of a collection of the Dietaries of most of the London hospitals. It is proposed to add those of the principal provincial institutions and of the London workhouse infirmaries, and thus complete a collection which must prove of great value for ready reference to secretaries and other officials of medical charities.

The following papers will be read and discussed at the General Meetings of The Hospitals Association held during the session 1887-88 :—1887 : November 9, "On Hospital Construction," by Keith D. Young, Esq., F.R.I B A. ; December 14, "On the Registration of Nurses," by J. C. Steele, Esq., M.D. 1888 : January 11, "On the Amelioration of the Present Condition of Midwives," by J. H. Aveling, Esq., M.D. ; February 8, "On the Relation of the Medical School to the Hospital," by T. Gilbart-Smith, Esq., M.D. ; March 14, "On Contributions by Patients in relation to the Financial Condition of London Hospitals," by W. Burdett-Coutts, Esq., M.P. ; April 11, "On the Origin, History, Work, and Present State of Metropolitan Lying-in Hospitals," by Thomas Ryan, Esq.

Sir Andrew Clark presided, in the absence of Mr. Jonathan Hutchinson, at the dinner inaugurating the new buildings at the London Hospital. He spoke eloquently on a congenial topic—the history and work of the great medical school, and on the abundant proofs it continued to give of its adaptability to the changing circumstances of the time. He demanded special honour for the school, in that it was one of the first, if not the first, to use hospital cases for the purposes of clinical teaching. It is one of Sir Andrew Clark's persistent contentions that the general hospitals deserve the support of the public quite as much on the ground of their incalculable value as scientific workshops, where medical men learn the practical part of their art, as on account of the important functions they perform as charitable institutions. If the public were a little more open-eyed and intelligent, the force of Sir Andrew's contention would be universally admitted, to the great increase of the prosperity of the hospitals.

"Voluntaries"—for an East London hospital, is a new book published by Mr. D. Stott. Among the contributions is one by Lord Lytton, beginning, "How fleas are put to death we all do know." One of the best poems in "Underwoods" finds a place. In a sonnet the Bishop of Bedford describes how, when he was "weary with the strife and din," and wanted to "flee from out the fray," a little stranger child came to him, climbed upon his lap, and comforted him. The most melancholy page is the record of the weights of 250 patients at the Children's Hospital. The contrast of the weights as they are with the weights as they should be is heartbreaking.

At the Church Congress at Wolverhampton last week, the Rev. Canon Isaac Taylor read a paper, in which he affirmed the superiority in many respects of Mahometanism to Christianity as a civilising agency for the lower races. The Christian faith, in itself, was higher and more noble than any other : too noble indeed for the less advanced races of the East. The more immediately practical and comprehensible faith of Islam could be readily apprehended by uneducated tribes, and was a great advance on their former superstitions. Said the Rev. Canon : "It replaced monkliness by manliness. It gave hope to the slave, brotherhood to mankind, and recognition to the fundamental facts of human nature."

THE following vacancies are announced this week, the amount of the salary being placed in each case after the name of the institution :—

**Resident Medical Officers.**

Birmingham Borough Asylum. Clinical Assistant.—No salary.
Cumberland Infirmary, Carlisle. Assistant House-Surgeon.—£40.
Hospital for Sick Children, Great Ormond Street. Junior Resident Officer.—£50.
National Consumption Hospital, Bournemouth. Resident Medical Officer.—£150.

**Non-resident Medical Officers.**

Royal Berks Hospital, Reading. Physician.
Sheffield Hospital. Honorary Physician.
Taunton Hospital. Honorary Surgeon.

**Matrons.**

Stafford Infirmary.—£60.
West Bromwich District Hospital.—£40.

**Trained Nurses.**

Bradford Infirmary, Yorkshire. Night; fully trained.—£25, uniform.
Bristol Hospital for Sick Children. Surgical and Medical. Two.
Chester Diocesan Deaconess' Institution. Two fully-trained.—£30 Church of England.
Kent Nursing Institution. Three.
Newport Infirmary, Monmouth. Night.—£25, uniform. Also Assistant and Probationer.
Norwich Nurses' Home. Two.
Stanley Hospital, Liverpool. Three years' training, £22.

---

THERE has been a little quarrel at Falkirk about a football match played on behalf of the proposed cottage hospital. Quarrels are not very uncommon at football matches, but this is the first we remember to have heard of at a match played for charitable purposes, and we hope it will be the last.

---

NEW experimental laboratories have been opened at Edinburgh, in connection with the Royal College of Physicians, under the superintendence of Dr. Sims Woodhead. Dr. Woodhead has established for himself a high reputation as a scientific worker in the fields of biology and pathology, and there is every reason to hope that under his able supervision the new laboratories may become centres of much valuable original work.

---

AN income of £5,245 18s. 9d., with an expenditure of £5,243 1s. 10d., would have satisfied Mr. Micawber's conditions of financial happiness. The managers of the West of Scotland Convalescent Seaside Homes are in the happy position indicated by this statement. Their institution belongs to that very small number of similar associations which can report a slight increase in ordinary subscriptions, and also from churches, Bible-classes, Sabbath schools, and foundry-boys' meetings. During the past year, income, expenditure, and work all show progress, and the committee are able to begin a new year with a balance of £2 16s. 11d. in the bank.

---

SMOKING is by no means universally indulged in on the Episcopal Bench. Of the thirty-four eminent dignitaries who now occupy that exalted position, more than half are non-smokers. The Bishop of Bath and Wells has not used tobacco in any form for more than fifty years. The Bishop of Worcester smokes neither pipe nor cigar, and never did. The Bishop of St. Albans has never smoked in his life. The Bishop of Durham is a non-smoker, but does not wish the statement to convey any sympathy with, or feeling of necessity for a league against tobacco. The Bishop of Gloucester and Bristol was a moderate smoker until 1860, but when he found that the benefit he thought he had derived from it ceased, he gave it up. The Bishop of Oxford is not and never has been a smoker. "He believes that in time, in purse, in health, and in cleanliness he has been the gainer." The Bishop of Liverpool is a non-smoker, and entirely approves of the Anti-Narcotic League. King James' "Counterblast" is not without its modern counterpart.

THE meeting of The Hospitals Association, at which the National Pension Fund scheme was explained by Mr. Burdett, attracted a crowded and representative audience on Wednesday, several hundreds being present. Sir Andrew Clark made an ideal president, and his strong personal interest in and support of the scheme is of great importance and encouragement at this juncture. A full account of the proceedings will be published next week.

---

THE hope we ventured to express, that the epidemic of scarlet-fever had possibly reached its maximum, has not been realised. Cases continue to pour into the hospitals of the Asylums Board in increasing numbers. Most of the hospitals are full, or nearly so, and the convalescent institutions are in the same condition. There is probably yet no serious reason for alarm, since it cannot be doubted that the readiness of the public to take advantage of the Board's hospitals, and the vigilance of all grades of sanitary officials, combine to swell the numbers removed from home for treatment beyond all previous experience. With the colder weather, especially if the atmosphere should be clear and frosty, we may hope for some amelioration within a measurable period.

---

MR. JAMES CUNNINGHAM, who died some years ago, left £1,000 as a legacy to the Blackburn and East Lancashire Infirmary. For some unexplained reason the money was not paid over to the authorities at the time of Mr. Cunningham's death. It has now been received and placed to the credit of the endowment fund.

---

THE cost of defending an action brought by Mr. Ensor against some of the members of the committee of the Cardiff Infirmary has been, by resolution of the committee, defrayed out of the Infirmary funds. A well-attended meeting of the workmen employed in the Bute yards unanimously passed a resolution affirming their strong disapproval of such a use of charitable resources.

---

MR. ALEXANDER STEWART, in his "Reminiscences of Dunfermline," tells how a certain gravedigger of the "linen" town used to complain that the constant recollection of his peculiar calling operated as a check to his natural affability. "'Deed man"—he would say—"I'm feared to speer at onybody hoo they are, in case they micht think I was wearying on them, or even ower a dram to say, 'My service to ye!'"

---

JOSEPH MARTIN, aged fifty, a carrier, committed suicide at Lansfield Street, Queen's Park, last week. He had suffered severely from rheumatic gout for nearly a year, and was quite unable to work. His dread of the approaching winter, with his inability to earn his livelihood, had impelled him to the melancholy act.

---

THE Earl of Yarborough presided at the annual meeting of the subscribers to the Grimsby and District Hospital at the town-hall on Monday. The honorary secretary, Mr. E. Bannister, read a report, which showed that the number of patients continued to increase steadily, and that the work done had been in every respect satisfactory during the past year. The financial affairs of the hospital are by no means so prosperous. There has been a diminution in the income for some time. Last year the deficit amounted to nearly £200, which, added to former debts, leaves a balance against the hospital of £900. A portion of the amount collected for the Jubilee fund has been given towards the reduction of this adverse balance.

An effort has been progressing for some time in Islington, having for its object the raising of £20,000 for the completing and furnishing of a wing of the new Great Northern Central Hospital, to be called the Jubilee Wing. It is much to be feared that the £20,000 will not be reached this year. A first instalment of £2,000 has been paid to the committee for the purpose of meeting liabilities now due. The fund is still open.

---

Mr. Arthur Pawson, barrister-at-law, was found dead in his room one morning last week. At the inquest it was said he had suffered from neuralgia, toothache, and a disordered liver, and could not sleep. He had been in the habit of using laudanum and chloral, and beside his dead body was found a glass measure containing laudanum and water. The medical evidence was to the effect that death had resulted from laudanum, about five teaspoonfuls having been taken. The verdict was one of "death from misadventure."

---

A Russian adventuress, known by the nickname of the "Golden Hand," has been condemned by the tribunal of Moscow to transportation for life. This extraordinary woman, who is described as being remarkably handsome, has been married no fewer than sixteen times.

---

At the last meeting of the committee of the Royal Portsmouth Hospital, the Vicar of Portsmouth (the Rev. E. P. Grant) announced that he was compelled to resign the office of chairman, as, owing to the state of his health, he felt unequal to the responsibilities and work connected with the chairmanship. He had been connected with the institution for fifteen years, and had seen the beds increased from fifty-six to seventy. He did not intend to sever his connection with the Committee of Management, and hoped to be often there. Admiral Sir Henry Chads, K.C.B., as the senior member of the committee, and the Admiral Commander-in Chief, Sir George Willes, K.C.B., bore testimony to the time and energy which the Vicar had devoted to the affairs of the institution, and expressed a hope that he might reconsider his decision. It is understood that the Mayor (Sir William King) will be asked to accept the office.

---

Miss Louisa Twining writes :—"With regard to your remarks on my letter, will you allow me to explain that my statements are the result of many years' observation of country life, all over England; but this year's experience was gathered in a north midland county, and not in the south? Had I seen the labourers feeding at the farmers' tables in Lincolnshire or Yorkshire, I do not think it would have in the least affected my theory as regards family life in towns and villages. The range of my observations cannot possibly be known to readers, because I do not name it; and I still repeat that the three causes I named are the chief reasons of weak health, and I am certain they could be taught to wash, air their rooms and clothes, and to cook at least the vegetables out of their own gardens, and make soup as well as their French neighbours. I may add just one case of a woman, comfortably off, living in an ideal lovely spot, with a clear mountain stream of purest water flowing near; there she had lived for forty years, she told me, but was "never well," had no appetite, and had buried seven children! A more desponding tale could hardly have been told in one of our worst East-end slums. I believe I have helped to point the way to a cure, by urging the teaching and information given by our two chief Health Societies, The Ladies' Sanitary Association, and National Health Society, both in Berners Street, W."

Thomas Harrison, eleven years of age, and Robert Audley, six, were charged at Bootle with stealing fivepence-halfpenny from a collection cage fixed to a lamp-post on behalf of the Hospital Saturday Fund. These juvenile thieves, armed with match-boxes, seem to have attempted to set fire to the collecting cage. By that or other means they succeeded in abstracting the sum in question. Several other boys at Bootle were brought up on a similar charge. In every case where the offence was proved, six strokes of the birch were ordered to be administered.

---

The Toys of *Truth* are already in course of manufacture for Christmas, and many are the boys and girls, and even infants, who would be glad to remain in their several hospitals until the dawn of the happy morning of distribution. 14,000 were found necessary last year; this year 20,000 have already been asked for. Mr. Labouchere is the *Deus ex machina* of the toy-shop, and in that region, at any rate, his oracle is understood to be the most perfect and sublimed distillation of wisdom and benevolence. He asks for prompt and immediate generosity on the part of his Toy constituents. In view of the large increase in the number of exhibits, the directors of Willis's Rooms have most liberally placed their large ball-room at his disposal for the exhibition of both bought and home-made toys. The show will be opened on Monday, December 19th, and remain open for two full days. This is the first whiff of Christmas air we have had, and it is so bright and bracing as almost to console us for the rapidly waning summer. Would that all human affairs could be made as "pleasant all round" as Mr. Labouchere's Annual Christmas Toy Show.

---

A deputation of 150 people assembled on Saturday in the street leading to the Local Government Board Offices. They carried a black flag with the words, "We must have work or bread." Mr. A. D. Adrian, Assistant Secretary to the Local Government Board, intimated his readiness to see two or three delegates. A resolution was submitted to Mr. Adrian, which it was stated had been passed. The resolution set forth—"That this meeting of homeless men and women claims the right to live by honest labour, and therefore calls upon the Government to immediately start relief-works, at a reasonable rate of wage; and, failing this, further expresses its firm determination to use every means in its power to put a stop to the misery and suffering of thousands of honest, hard-working men and women, and their children, as witnessed nightly in Trafalgar Square." The persons assembled then marched through several streets in procession, accompanied by policemen, mounted and on foot. They expressed their determination to parade the streets day by day until relief in the form of work or otherwise should be found. This is a most moving incident. Every man and woman who can think should feel it an imperative duty to use his own personal influence in procuring work, or other relief, for these starving people without delay. It must be remembered that Government officials are merely public servants. They cannot be expected to take the initiative unless they are prompted and guided by the reason and sympathy of unofficial men and women. The long and chilling nights of winter are fast approaching, and it is heart-breaking to think of aged people and young children, half-clothed, and starving with cold and hunger, lying all night on the bare ground, with nothing but the sky overhead. Now is the time for all who sympathise with those unhappy and destitute people, to unite together for a great and common effort to find them, at the least, shelter and food.

